# Stepping toward a Macaque Model of HIV-1 Induced AIDS

**DOI:** 10.3390/v6093643

**Published:** 2014-09-25

**Authors:** Jason T. Kimata

**Affiliations:** Department of Molecular Virology and Microbiology, Baylor College of Medicine, One Baylor Plaza, Mail Stop BCM385, Houston, TX 77030, USA; E-Mail: jkimata@bcm.edu; Tel.: +1-713-798-4536; Fax: +1-713-798-4435

**Keywords:** HIV-1, *Macaca nemestrina*, pathogenesis, AIDS, innate restriction

## Abstract

HIV-1 exhibits a narrow host range, hindering the development of a robust animal model of pathogenesis. Past studies have demonstrated that the restricted host range of HIV-1 may be largely due to the inability of the virus to antagonize and evade effector molecules of the interferon response in other species. They have also guided the engineering of HIV-1 clones that can replicate in CD4 T-cells of Asian macaque species. However, while replication of these viruses in macaque hosts is persistent, it has been limited and without progression to AIDS. In a new study, Hatziioannou *et al.*, demonstrate for the first time that adapted macaque-tropic HIV-1 can persistently replicate at high levels in pigtailed macaques (*Macaca nemestrina*), but only if CD8 T-cells are depleted at the time of inoculation. The infection causes rapid disease and recapitulates several aspects of AIDS in humans. Additionally, the virus undergoes genetic changes to further escape innate immunity in association with disease progression. Here, the importance of these findings is discussed, as they relate to pathogenesis and model development.

## 1. Introduction

Primate lentiviruses are highly adapted to replication in their native hosts but have limited capacity to infect and spread to new species [1]. In particular, HIV-1 is restricted to humans and chimpanzees, the host from which it was derived, impeding the development of a robust animal model of HIV-1 infection and disease that could be used for testing vaccine, therapeutic, and curative strategies. Over the past twelve years, studies have demonstrated that host species have evolved innate restriction factors to block lentiviral infection, and the targeted viruses have evolved countermeasures to evade the effects of the restriction factors. For example, apolipoprotein B mRNA-editing, enzyme-catalytic, polypeptide-like 3G (APOBEC3G), tetherin (BST-2 or CD317), tripartite motif-containing protein 5α (TRIM5α), and TRIM5-cyclophilin A (TRIMcyp) fusion proteins interfere with replication at different stages of the viral life cycle [2,3,4,5,6]. Activities of two of these proteins, APOBEC3G and tetherin are inhibited by the virally encoded proteins Vif and Vpu, respectively, while mutations in capsid enables HIV-1 to escape recognition by TRIM5α. Interestingly, the species-specific tropism of primate lentiviruses correlates with evasion of TRIM5α or functional activity of the viral antagonists against APOBEC3G or tetherin [7,8,9,10,11,12,13]. These data suggest that innate immunity is a formidable defense against the transmission of lentiviruses into new species.

## 2. Engineering Macaque-Tropic HIV-1

As an alternative way to test this hypothesis, several groups have engineered HIV-1 to be resistant to restriction factors of Asian macaque species, examined replication in CD4 T-cells of these species, and challenged the respective hosts to determine if the “macaque-tropic” viruses could establish persistent infection and cause AIDS. Given the utility of rhesus macaques (RM, *Macaca mulatta*) for vaccine testing and studies of pathogenesis, initial experiments focused on developing macaque-tropic HIV-1 for replication in this species. HIV-1 clones with substitutions of SIVmac capsid or capsid mutations and the SIVmac *vif* gene (macaque-tropic HIV-1) were constructed to overcome inhibition mediated by the known restriction factors, rhesus TRIM5α and APOBEC3G [14,15]. However, these clones only replicated well *in vitro* after multiple rounds of passage, and they have not yet proven to establish persistent infection in RM. CD4 T-cells of a related macaque species, *Macaca fascicularis* (cynomolgus monkeys, CM) are also susceptible to macaque-tropic HIV-1 clones, but viral replication is quite poor and easily controlled in host animals [16]. Perhaps contributing to the difficulty of adapting macaque-tropic HIV-1 to either RMs or CMs is that both species are polymorphic for TRIM5α alleles and TRIMcyp [17,18], which can interfere with infection [19,20,21].

The most promising results with macaque-tropic HIV-1 clones have been achieved using *Macaca nemestrina* (pigtailed macaques, PTM) as an experimental host. While genetically and immunologically less well characterized than the RM, PTM arguably offered greater potential as a possible model. Key early findings were initially made by Agy *et al.* [22], who had shown that PTM are susceptible to HIV-1, although infection is not persistent, does not cause disease, and HIV-1 is not able to adapt after *in vivo* passaging [23]. Subsequent studies also demonstrated that unlike RM cells, PTM cells are easily transduced with HIV-1 based vectors [24]. The greater susceptibility is due to the absence of TRIM5α or a TRIM5-cyclophilin A fusion protein capable of blocking HIV-1 infection [17,18,25]. Even more, the mutation preventing TRIM5α expression appears to be homozygous in PTMs. Thus, there is one fewer innate restriction factor for HIV-1 to evade in PTMs. Indeed, a simple replacement of *vif* with that from SIVmac or SIVmne alone is sufficient for HIV-1 to replicate robustly in CD4 T-cells of PTMs [26,27]. Infection of PTMs with modified HIV-1 clones results in an initial spike in viral load, strong antibody responses, and low-level replication for at least two years, but the infections are eventually controlled [26,27,28]. Why the viruses do not replicated more robustly is not entirely clear. Limited replication could be in part due to virus-specific CD8 T-cell responses, as administration of an anti-CD8 antibody can cause a transient rebound in viral load. Neutralizing antibodies may also contribute to control. Furthermore, unlike pathogenic SIVmne, replication of these macaque-tropic HIV-1 clones in PTM T-cells may be suppressed by interferon alpha (IFNα), suggesting these viruses do not fully escape the type I IFN response that occurs during acute infection and that further adaptation may be required for greater replication in the PTM host [29,30].

## 3. Passaging Macaque-Tropic HIV-1 to Increase Pathogenicity

**Figure 1:**
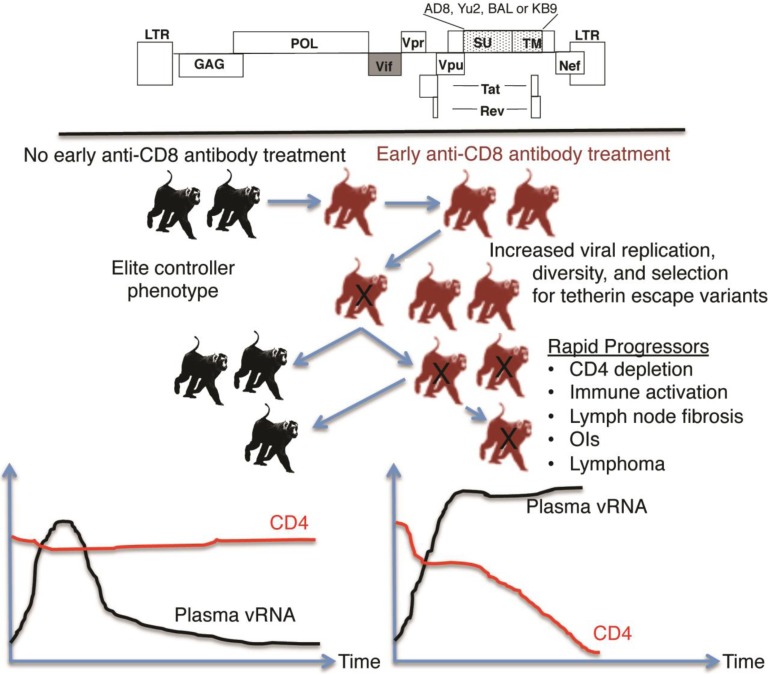
Selection of pathogenic macaque-tropic HIV-1. Pig-tailed macaques were inoculated with a mixture of macaque-tropic HIV-1 clones that contained the *env* genes of four different CCR5-tropic viruses in a NL4-3 backbone with a SIVmac239 *vif* replacement. Viruses were inoculated and passaged through pigtails that received anti-CD8 antibody treatment (red) or no treatment (black). Early CD8 cell depletion increased viral replication and emergence of pathogenic macaque-adapted HIV-1. Rapid disease progression (X) depended on CD8 depletion as pigtails that did not receive early anti-CD8 antibody treatment controlled the infecting virus.

Can passaging macaque-tropic HIV-1 in PTMs also increase replication fitness and pathogenicity? If past studies of SIV are an indication of what will happen, then macaque-tropic HIV-1 should eventually become more pathogenic, as *in vivo* passaging of SIV promotes the emergence of variants with increased replication fitness and pathogenicity [31,32]. In a recent study, Hatziioannou *et al*. serially passaged a mixture of CCR5-tropic macaque-tropic HIV-1s in PTMs (Figure 1). The macaque-tropic HIV-1 clones encoded different subtype B *env* genes in the background of HIV-1NL4-3. Among these *env* genes are two well-characterized variants, KB9 and AD8, which have been used successfully for the generation of pathogenic Env-SIV-HIV chimeras (Env-SHIVs). Given that previous experiments had demonstrated transient rebounds in plasma viral loads after depletion of CD8 cells, animals were treated with anti-CD8 antibodies in order to hasten adaptation to PTMs and outgrowth of pathogenic variants [33]. This method had been previously used to accelerate the emergence of pathogenic Env-SHIV chimeras [34,35,36]. CD8 depletion is believed to enable greater early viral replication and generate a larger pool of viral variants from which a virus with increased fitness is likely to emerge. Interestingly, with each passage of the macaque-tropic HIV-1 variants in PTMs treated with anti-CD8 antibodies, viral loads increased, and by the fourth passage of the viruses in PTMs, viral loads were sufficiently high in one animal to cause rapid progression to AIDS. Viral loads were also increased in the other animals, although there were no signs of disease in the one-year time frame of the experiments shown. The macaque-tropic HIV-1 *env* variant, AD8, in the virus mixture also became the predominant virus after the second passage, suggesting a selective advantage of this *env* for replication in PTMs.

To confirm the results, the adapted viral variants were passaged through two additional sets of PTMs. In each case, animals that received anti-CD8 antibodies at the time of inoculation demonstrated rapid loss of CD4 T-cells and very high viral loads. In some regard, pathogenesis in these animals resembled that of HIV-1 infection and recapitulates aspects of AIDS. For example, disease progression was associated with increased Ki-67 expression in T-cells, suggesting immune activation. There was also evidence of lymphoid fibrosis, opportunistic infections, and lymphoma. On the other hand, these animals were more similar to SIV-infected rapid progressor macaques [37]. CD4 T-cell decline was rapid, there were no anti-HIV antibody responses, and viral production was mainly from macrophages at the later stages of infection. Sequencing analysis of the *env* gene demonstrated evolving diversity of 2% from the dominant parental virus, AD8, *env* over the course of passaging. Additionally, the sequencing analysis of the envelope (*env*) gene revealed a 4 amino acid V3 deletion in about 50% of the viruses that was associated with a switch to CXCR4 coreceptor usage in one animal. The authors confirmed the switch by demonstrating a change in sensitivity of viruses pseudotyped with the parental AD8 Env and passage 5 Env V3 deletion mutants from maraviroc (CCR5 antagonist) to AMD300 (CXCR4 antagonist). Thus, like HIV-1 infection in humans, coreceptor switching can occur during macaque-tropic HIV-1 infection in association with disease progression.

While the macaque-tropic HIV-1 clones were designed to counteract APOBEC3 proteins of PTMs, they do not antagonize macaque tetherin because the HIV-1 Vpu protein does not commonly inhibit macaque tetherin function [11,13]. Interestingly, the authors found the emergence of an insertion at amino acid position 15 and a valine to glycine change at position 21 in the transmembrane domain of Vpu. Each mutation conferred a gain of function, enabling the HIV-1 Vpu to inhibit PTM tetherin-mediated suppression of virion release, demonstrating the importance of antagonizing tetherin for persistence in a non-native host. Additionally, these mutations occur in the same region of Vpu present in some Env-SHIVs that enhance pathogenicity and antagonize RM tetherin [38]. However, in the context of macaque-tropic HIV-1, the acquisition of the mutations alone were not sufficient to increase pathogenicity in the PTMs that did not receive anti-CD8 antibody treatment. These animals still retained their elite controller phenotype. On the other hand, it is important to note that the follow up period for the experiments was only about one year and that CD4 T-cell decline and disease in the absence of CD8 T-cell depletion may require infections of longer durations.

Why macaque-tropic HIV-1 variants appear to replicate well only after early CD8 T-cell depletion is not entirely clear. One reason may be that these viruses have limited ability to evade CTL responses because they cannot easily evolve escape mutations in CTL epitopes without losing significant replication fitness, or they may not efficiently downregulate PTM major histocompatibility class I antigens. The viruses may also need to evolve mutations that enable escape from neutralizing antibodies in order to further enhance replication fitness *in vivo*. Each of these mechanisms of evasion of adaptive immune responses has been shown to play an important role in persistent SIV infection of macaques [31]. Thus, it seems likely that macaque-tropic HIV-1 clones must also acquire similar mutations throughout the genome for increased replication and pathogenicity in the PTM host. Another possibility is that the viruses might require further adaptation to PTMs in order to evade other type I IFN-inducible innate restriction factors. A type I IFN resistant phenotype could be essential for robust viral replication during the acute stage of infection, depletion CD4 T-cells, and suppression of CD4 helper T-cell dependent responses. Finally, it may also be important to examine the contribution of natural killer cells (NK cells) and NK cell-mediated responses for controlling macaque-tropic HIV-1, as well as the ability of macaque-tropic HIV-1 to counteract and evade these responses.

Why the AD8 *env* emerged as the dominant allele is unknown. A possible explanation is that the AD8 Env has a greater ability to bind the PTM CD4 protein compared to the other HIV-1 Env proteins used, and therefore, it is able to outcompete those viruses. In support of this idea, previous studies with subtype B Env-SHIVs as well as a subtype A macaque-tropic HIV-1 have shown the emergence of variants with increased binding to macaque CD4 after *in vitro* passage on macaque CD4 T-cells or passage through animals *in vivo* [39,40,41], indicating that the HIV-1 *env* must adapt to macaque CD4 for efficient viral replication. Further studies of the AD8 *env* will be necessary in order to define the characteristics associated with its dominance and if *env* variants exhibiting similar phenotypes may also contribute to increased replication fitness of macaque-tropic HIV-1.

## 4. Concluding Remarks

Overall, the studies by Hatziioannou *et al.*, take an important step forward. Using an experimental infection model, they provide strong evidence for the impact innate restriction factors have on cross-species transmission of lentiviruses. Indeed, APOBEC3G, TRIM5, and tetherin dictate whether a virus can establish persistent infection and cause disease in a new non-native host. When considered in context of earlier infection studies of wild type HIV-1 infection of PTMs, innate restriction factors provide a formidable initial line of defense that may be very difficult to overcome, as wild type HIV-1 is unable to adapt to PTMs even after multiple rounds of *in vivo* passaging of virus. Thus, these studies support strategies for using innate restriction factors in gene therapy approaches for protecting cells from HIV-1. While many questions remain about the mechanisms underlying immunological control of macaque-tropic HIV-1, studying the infection of PTMs may provide insights relevant to elite control of HIV-1. Finally, the potential for causing AIDS in a macaque species with engineered HIV-1 opens the door for a model of HIV-1 pathogenesis that could be used for testing vaccines, antiretroviral treatment, and cure strategies that require specific targeting of HIV-1 proteins and sequences.
